# Single-session versus two-session placement of chest port and gastrostomy tube in patients with head and neck cancer: Is there any difference in the device-related early infection rates?

**DOI:** 10.1177/20584601211037234

**Published:** 2021-08-29

**Authors:** Philip Skummer, Katsuhiro Kobayashi, Mason Schoeneck, Jamynkumer Patel, Masoud Faridnia

**Affiliations:** 1 Department of Radiology, 5506 Medical College of Wisconsin, Milwaukee, WI, USA; 2 Department of Radiology, 12302 SUNY Upstate Medical University, Syracuse, NY, USA; 3 Department of Anesthesiology, 12297 New York University, NewYork, NY, USA; 4 Department of Medicine, 6889 Virginia Common Wealth University, Richmond, VA, USA

**Keywords:** infection < topics, gastrostomy, chest port

## Abstract

**Background:**

It is unknown whether placement of a chest port (port) and a gastrostomy tube (G-tube) in a single session increases the risk of the early device infections in patients with head and neck cancer (HNC) undergoing chemoradiation.

**Purpose:**

To compare the incidence of early (≤30 days) port and G-tube infections placed in a single session compared to two separate sessions in patients with HNC.

**Material and Methods:**

Between January 2012 and December 2019, 169 patients with HNC undergoing chemoradiation had a port and a G-tube placed in a single session (single-session group), while 25 had both devices placed in two separate sessions (two-session group) within 30 days of each other. The incidence of early device infections was compared between groups. Logistic regression analysis was conducted to determine if the number of sessions was a variable affecting device infections.

**Results:**

A total of 6 (3%) early port infections and 13 (6.7%) early G-tube infections were identified. The two groups did not significantly differ in the incidence of early port infections (3.0%, 5/169 and 4.0%, 1/25, *p* = 0.59) nor early G-tube infections (7.1%, 12/169 and 4.0%, 1/25, *p* = 1.0). The number of sessions for device placement was not a variable affecting overall device infections in logistic regression analyses (odds ratio: 1.24, 95% confidence interval: 0.20–7.82, *p* = 0.82) after controlling for potential confounding variables.

**Conclusions:**

The risk of early device infections in single-session placement appeared to be the same as two-session placement in patients with HNC undergoing chemoradiation.

## Introduction

Concurrent chemoradiation is the current standard of care for patients with locally advanced head and neck cancer (HNC). Chemoradiation produces clear survival benefits over radiation therapy alone, but the acute toxicity is increased by two- or three-fold.^1^ High-grade mucositis is the most prevalent acute toxicity, which can potentially result in malnutrition or dehydration during treatment.^2^ Additionally, locally advanced HNC may impinge on the esophagus or oropharynx causing dysphagia and disruption to oral intake. Patients typically need placement of an implantable chest port (port) to allow for delivery of chemotherapy agents and a gastrostomy tube (G-tube) to provide nutritional support and hydration throughout chemoradiation therapy. It would be convenient for the patients to have both a port and a G-tube placed at the same time (single-session placement). Single-session placement can avoid repeating the pre-procedural process such as nothing by mouth, modification of anticoagulant or antiplatelet medications, and two separate anesthesia encounters. Furthermore, patients with single-session placement could theoretically initiate treatment sooner compared to patients who have the two devices placed at different times (two-session placement).

Port and G-tube placements differ in the cleanliness of the procedure and confer different risk of infection. Port placements are classified as clean procedures, while G-tube placements are classified as clean-contaminated procedures according to the Society of Interventional Radiology (SIR) practice guidelines.^3^ There is a theoretical risk of cross-contamination between the bloodstream and the gastrointestinal tract during single-session placement, which could manifest as an early infection. No studies have investigated whether single-session placement would confer a higher risk of the device-related infections compared to two-session placement to the authors’ knowledge.

The purpose of this retrospective study was to compare the incidence of port and G-tube infections within 30 days of placement (early infections) between patients with HNC undergoing chemoradiation who had single-session placement of the two devices and those who had two-session placement. Furthermore, this study sought to determine if the number of sessions for the device placements could be a variable affecting the risk of early device infections in this patient population.

## Material and methods

### Patients

**Figure 1.**
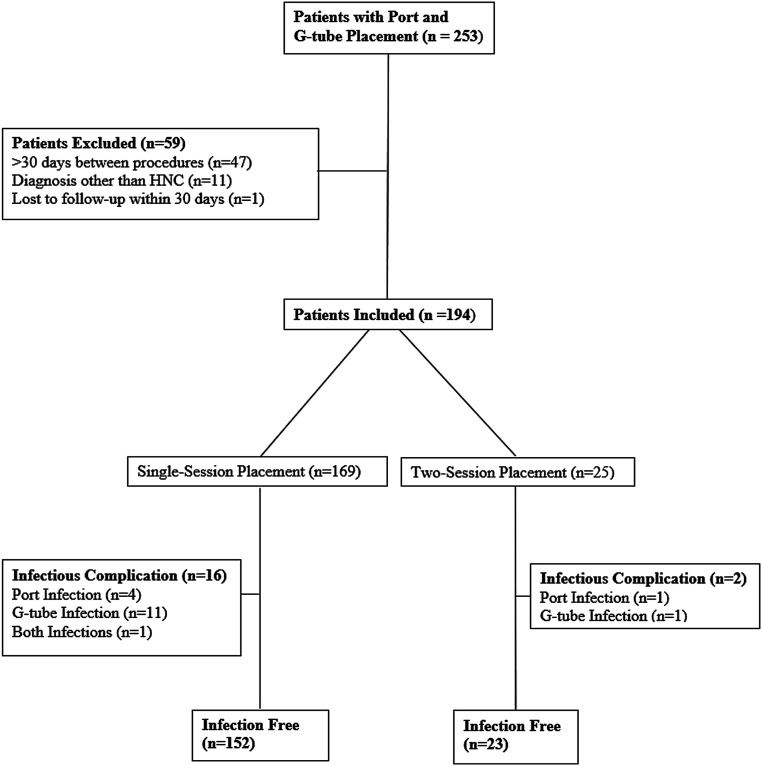
Flow chart of study population.

This single-center retrospective review complied with the Health Insurance Portability and Accountability Act and was approved by the Institutional Review Board. Patients who underwent port or G-tube placement by the Division of Interventional Radiology between January 2012 and December 2019 were identified by searching the picture archiving and communication system. A total of 253 patients underwent both port and G-tube placement during this time period. Patients with a diagnosis of HNC were included in the study if port and G-tube placements occurred within 30 days of one another. Patients were excluded if they were lost to follow-up within 30 days (*n* = 1), did not have a diagnosis of HNC (*n* = 11), or had a port and a G-tube placed greater than 30 days apart (*n* = 47). A flow chart outlining the inclusion and exclusion of patients is illustrated in Figure 1

.

The final study population included 194 patients; 169 patients had a port and a G-tube placed in a single session (single-session group), and 25 patients had the devices placed in two sessions (two-session group). All patients underwent subsequent chemoradiation therapy. The indication for all G-tubes was nutritional support, and the indication for all ports was chemotherapy infusion. Any device-related early infections were recorded through the review of the patients’ electronic medical records and imaging studies. Device-related early infections were classified as major or minor complications.^4^ Additionally, port infections were subcategorized as a port-site infection or a port-associated blood stream infection (PABSI).

### Technique

The patients’ coagulation parameters and complete blood cell counts were evaluated prior to port or G-tube placement. Coagulopathy (international normalized ratio >1.5) or severe thrombocytopenia (platelet count <50,000/μL) was typically corrected prior to the procedure, or the procedure was delayed until these laboratory counts were improved. Device placement was generally avoided in patients with severe neutropenia (absolute neutrophil count (ANC) <500 cells/μL) because of the risk of infection.^5^ All patients were given prophylactic intravenous antibiotics prior to device placement, either 1 gram of cefazolin or 600 milligrams of clindamycin for patients with a penicillin allergy.

The procedures were conducted by 6 interventional radiologists with 2–25 years (median 6 years) of experience with venous and abdominal interventions. For single-session placement, both surgical sites underwent sterile preparation and the patient’s whole body was then covered with a surgical drape. A window of approximately 30 square centimeters was created in the sterile surgical area for each procedure. All port placements preceded G-tube placements. The port site was covered with a sterile towel immediately after skin closure. The operators changed their sterile gloves and gowns between the device placements. The same sterile back table was used for both procedures.

### Port placement

All ports were placed in interventional radiology suites using the standard fashion.^6^ The right internal jugular vein was preferentially accessed under ultrasound guidance; however, the left internal jugular vein was used if the right internal jugular vein was thrombosed or if the patient’s right upper chest had previously been irradiated. The skin incision of the port pocket was closed with interrupted subcutaneous 2-0 polyglactin sutures and a running subcuticular 4-0 polyglactin suture (Vicryl, Ethicon Inc., Somerville, NJ, USA). Topical skin adhesive (Dermabond, Ethicon Inc., Somerville, NJ, USA) and reinforced adhesive skin closures (Steri-Strips, 3M, St. Paul, MN, USA) were applied over the port incision and jugular venous access site. Both were then covered with adhesive wound dressings (Covaderm, DeRoyal, Powel, TN, USA). Either a single lumen (Dignity^®^ CT Ports, Medcomp, Harleysville, PA, USA) or double lumen port (Deltec^®^, Port-A-Cath^®^, Smith Medical, St. Paul, MN, USA) was implanted according to the referring physician’s request.

### Fluoroscopy-guided gastrostomy

Ultrasound was used to mark the caudal margin of the liver prior to G-tube placement. Patients were not routinely administered oral barium to opacify the transverse colon. After adequate insufflation of the stomach with air through a nasogastric tube, a gastrostomy site was chosen below the costal margin, above the transverse colon, and to the left of midline. Two or 3 T-fasteners (SAF-T-Pexy; Kimberly-Clark, Roswell, GA, USA) were deployed around the expected gastrostomy site before an 18-gauge needle was inserted into the stomach under fluoroscopic guidance. After serial dilatation of the tract over a guide wire, an 18 French gastrostomy tube (Kimberly-Clark, Roswell, GA, USA) was introduced into the stomach through a 22 French peel-away sheath. Contrast was injected through the G-tube to confirm intraluminal placement and the anchoring balloon was inflated with saline.

### Definitions

The thresholds used to define laboratory abnormalities are as follows: leukopenia as white blood cell (WBC) <3500 cells/μL, leukocytosis as WBC >11,000 cells/μL, neutropenia as ANC <1500 cells/μL, neutrophilia as ANC >7000 cells/μL, and hypoalbuminemia as serum albumin level <3.5 mg/dL. A port-site infection was defined as erythema of the skin over either the port or subcutaneous catheter as well as purulent drainage from the port pocket; site infections did not require a positive culture.^7,8^ Criteria that defined a PABSI included a recognized pathogen from at least one blood culture or a commensal organism from at least two blood cultures; and the patient had at least one sign or symptom of systemic infection (such as fever (temperature >38^o^C), tachycardia (heart rate >90 beats per minute), tachypnea (respiratory rate >20 per minute), or hypotension).^7^ These cultures or signs/symptoms could not be attributed to an infection at another site in the patient;^7,8^ patients only presenting with fever did not have their ports removed.

### Statistical analysis

Statistical analyses were conducted using SPSS Statistics for Windows, Version 25.0 (IBM Corporation, Armonk, New York). The baseline patient information, device characteristics, and incidence of device infections were compared between the groups. For categorical variables, a Pearson’s chi-square test or two-sided Fisher’s exact were used. For normally distributed and non-normally distributed continuous variables, a two-sided Student’s t-test and a Mann–Whitney U test were used, respectively. Logistic regression analysis was conducted to determine if the number of sessions was a variable affecting overall device infections. Patient or device characteristics that were significantly different between the two groups were included in the logistic regression analysis. A *P*-value of <.05 was considered statistically significant.

## Results

The baseline patient characteristics are shown in Table 1. The median patient age was 61.0 years (range, 30–85 years). There was a male sex predominance (73.2%). The most common location of HNC was the oropharynx (57.2%), followed by the larynx (17.5%) and oral cavity (11.3%). The median serum albumin at the time of port placement was significantly lower in two-session group (4.10 g/dL) compared to the single-session group (4.30 g/dL) (*p* = 0.04). The median serum albumin at the time of G-tube placement was also significantly lower in two-sessions group (4.00 g/dL) compared to the single-session group (4.30 g/dL) (*p* = 0.001). There were significantly more patients with leukopenia in the two-session group (12%) compared to the single-session group (1.2%) (*p* = 0.02). The device characteristics are shown in Table 2. Double lumen ports were primarily placed (77.3%). Most patients underwent port placement as an outpatient (84.5%); however, the two-session group had significantly more ports placed as an inpatient (36%) compared to the single-session group (12%) (*p* = 0.01). The median time between port and G-tube placements in the two-session group was 12 days (range, 1–29 days).

**Table 1.** Patient characteristics.

Characteristic	All patients (*n* = 194)	Single session group (*n* = 169)	Two sessions group (*n* = 25)	*p* Value
Age (Years)^*^	60 (13)	61 (13)	55 (12)	0.31
Range	30–85	30–85	32–74	—
Sex^†^				
Male	142 (73.2)	126 (74.6)	16 (64)	0.27
Female	52 (26.8)	43 (25.4)	9 (36)	—
Cancer location^†,‡^	—	—	—	0.69
Oropharynx	104 (53.6)	92 (54.4)	12 (48)	—
Larynx	31 (16.0)	28 (16.6)	3 (12)	—
Oral cavity	20 (10.3)	17 (10.1)	3 (12)	—
Hypopharynx	7 (4)	7 (4)	0 (0)	—
Nasopharynx	6 (3)	5 (3)	1 (4)	—
Maxillary sinus	2 (1)	1 (0.6)	1 (4)	—
Multiple sites	9 (5)	7 (4)	2 (8)	—
Unknown primary	15 (7.7)	12 (7.1)	3 (12)	—
Tracheostomy present^†^	19 (9.8)	15 (8.9)	4 (16)	0.28
Diabetes mellitus^†^	43 (22.2)	38 (22.5)	5 (20)	0.78
Lab values at port placement				
ANC (x1000 cells/μL)^*,§^	5.1 (2.7)	5.1 (2.6)	4.8 (3.7)	0.25
Range	1.0–13.9	1.0–13.9	2.1–11.5	—
Neutropenia (ANC <1500/μL)^†,§^	1 (0.5)	1 (0.6)	0 (0)	1.0
WBC (x1000 cells/μL)^*,**^	8.0 (3.1)	8.0 (2.9)	7.4 (3.0)	0.21
Range	3.1–23.1	3.1–23.1	4.3–13.5	—
Leukopenia (WBC <3500/μL)^†,**^	2 (1)	2 (1)	0 (0)	1.0
Albumin (g/dL)^*,††^	4.3 (0.5)	4.3 (0.5)	4.1 (0.6)	0.04^†††^
Range	2.4–5.1	2.4–5.1	2.6–4.6	—
Hypoalbuminemia (Alb <3.5g/dL)^†,††^	7 (3.6)	6 (3.6)	1 (4)	0.60
Lab values at G-tube placement				
ANC (x1000 cells/μL)^*,‡‡^	5.1 (2.8)	5.1 (2.6)	3.8 (4.0)	0.12
Range	1.0–13.9	1.0–13.9	1.8–11.0	—
Neutropenia (ANC <1500/μL)^†,‡‡^	1 (0.5)	1 (0.6)	0 (0)	1.0
WBC (x1000 cells/μL)^*,§§^	7.7 (3.3)	8.0 (2.9)	5.9 (5.9)	0.11
Range	2.9–23.1	3.1–23.1	2.9–15.0	—
Leukopenia (WBC <3500/μL)^†,§§^	5 (2.6)	2 (1.2)	3 (12)	0.02^†††^
Albumin (g/dL)^*,***^	4.2 (0.5)	4.3 (0.5)	4.0 (0.2)	0.001^†††^
Range	2.4–5.1	2.4–5.1	2.7–4.6	—
Hypoalbuminemia (Alb <3.5g/dL)^†,***^	9 (4.6)	6 (3.6)	3 (12)	0.11

* Values are presented as median (interquartile range).

† Values are presented as number (percent column total).

‡ Some patients had cancer in two locations.

§ Missing *n* = 9.

** Missing *n* = 5.

†† Missing *n* = 34.

‡‡ Missing *n* = 7.

§§ Missing *n* = 4.

*** Missing *n* = 31.

††† Significance at the .05 level.

ANC: absolute neutrophil count; WBC: white blood cell count.

**Table 2.** Device characteristics.

Characteristic	All patients (*n* = 194)	Single session group (*n* = 169)	Two sessions group (*n* = 25)	*p* Value
Timing of device placement^*^				
Port and G-Tube together	169 (87.1)	169 (100)	0 (0)	—
G-tube placed first	8 (4.1)	0 (0)	8 (32)	—
Port placed first	17 (8.8)	0 (0)	17 (68)	—
Time between port and G-Tube placements (Days)^†^	0 (0)	0 (0)	12 (21)	—
Range	0–29	0–0	1–29	—
G-tube first (Days)	—	—	5 (24)	—
Port first (Days)	—	—	14 (14)	—
History of prior port^*^	1 (0.5)	1 (0.6)	0 (0)	1.0
Port lumens^*^	—	—	—	—
Single lumen	44 (22.7)	41 (24.3)	3 (12)	0.21
Double lumen	150 (77.3)	128 (75.7)	22 (88)	—
Port placement^*^	—	—	—	—
Outpatient	164 (84.5)	148 (87.6)	18 (72)	0.01^‡^
Inpatient	30 (15.5)	21 (12.4)	9 (36)	—

* Values are presented as number (percent column total).

† Values are presented as median (interquartile range).

‡ Significance at the .05 level.

A total of 6 (3.0%) patients developed an early port infection. The summary of port infections is shown in Table 3. There was no significant difference in incidence between the single-session group and the two-session group (3.0%, 5/169 and 4.0%, 1/25, *p* = 0.59). One patient in each group developed a PABSI that was treated with intravenous antibiotics and port removal. Both blood cultures grew *Staphylococcus aureus*; one was methicillin resistant and the other was methicillin sensitive. Four patients developed a site infection in the single-session group; one patient was hospitalized and received intravenous antibiotics, while the others were treated as an outpatient with a course of oral antibiotics.

**Table 3.** Infection categories.

	All patients (*n* = 194)	Single session group (*n* = 169)	Two session group (*n* = 25)	*p* Value
Any infectious complication	18 (9.3)	16 (9.5)	2 (8.0)	1.0
Location of port infection				
Site infection	4 (2.1)	4 (2.4)	0 (0)	1.0
PABSI	2 (1.0)	1 (0.6)	1 (4.0)	0.24
Type of port infection				
All port infection	6 (3.1)	5 (3.0)	1 (4.0)	0.59
Major infection	3 (1.5)	2 (1.2)	1 (4.0)	0.34
Minor infection	3 (1.5)	3 (1.8)	0 (0)	1.0
Type of G-Tube infection				
All G-Tube infection	13 (6.7)	12 (7.1)	1 (4.0)	1.0
Major infection	2 (1.0)	2 (1.2)	0 (0)	1.0
Minor infection	11 (5.7)	10 (5.9)	1 (4.0)	1.0

Values are presented as number (percent column total).

PABSI: port-associated blood stream infection.

A total of 13 (6.7%) patients developed an early G-tube infection. The summary of G-tube infections is shown in Table 3. There was no significant difference in incidence between the single-session group and the two-session group (7.1%, 12/169 and 4.0%, 1/25, *p* = 1.0). Two (1.0%) patients in the single-session group developed a major infection requiring treatment with intravenous antibiotics. One patient ultimately developed *Streptococcus mitis* bacteremia. Both patients’ ports remained in place without signs of infection. All other G-tube infections were peristomal infections that were considered minor complications. These peristomal infections were managed with topical antibiotics and/or oral antibiotics. One patient in the single-session group received a single dose of intravenous antibiotics and removal of the T-fasteners, in addition to topical antibiotics.

Overall, 18 (9.3%) patients developed an early device (port or G-tube) infection. One patient in the single-session group developed both port and G-tube infections within 30 days of placement. There was no significant difference in incidence between the single-session group and the two-session group (9.5%, 16/169 and 8.0%, 2/25, *p* = 1.0). Logistic regression analysis revealed that single-session placement was not a significant variable affecting overall early device infections after controlling for inpatient port placement as well as serum albumin and leukopenia at the time of G-tube placement (odds ratio: 1.24, 95% confidence interval: 0.20–7.82, *p* = 0.82).

## Discussion

One of the most important goals of modern hospital hygiene and infection control is to reduce infection rates in sterile surgical procedures. The risk of infection varies among the surgical procedures depending on multiple factors such as local wound condition, the patient’s immune status, and the degree of contamination of the surgical site.^9^ Patients with head and neck cancer undergoing chemoradiation are a special subset of patient population at risk for postoperative wound infection due to potential poor nutritional status and immunosuppressive state secondary to underlying malignancy and oncologic treatment such as chemoradiation. Although port and G-tube infections in patients in head and neck cancer have been extensively investigated respectively, the risk of postprocedural infections of both devices, especially according to the number of sessions (single-session vs two-session placement), has not been investigated to the best of the author’s knowledge. An increased risk of port infections could be of particular concern when the two devices are placed in a single session since G-tube placements are clean-contaminated procedures while port placements are clean procedures.^3^ This study demonstrated no statistical difference in the incidence of early port, G-tube, or overall device infections between the single-session and two-session groups (*p* = 0.59, 1.0, and 1.0, respectively). In accordance with this result, the number of sessions for the device placements was not a variable affecting overall device infections in multivariate regression analyses (*p* = 0.82). Overall port infection rates in patients with HNC are reported to be higher, ranging between 5.8% and 8.5%,^10–12^ possibly related to local radiotherapy and tracheostomy^13,14^ compared to the infection rates reported in general cancer population ranging between 0.7% and 1.1%.^15–17^ The early port infection rates in the single-session group (3.0%) and two-session group (4.0%) are similar to the rate reported by Bos et al.^12^ who observed an early port infection rate of 3.3% in patients with HNC; this was significantly higher compared to the rate of 1.3% in their control group with non-HNC.

A breach in sterile technique during port placement has been attributed as a cause of early port infections.^12^ Inpatient placement,^16^ neutropenia,^15^ and serum albumin at the time of placement^8^ have also been reported to be risk factors for early port infections. Among these risk factors, inpatient placement (*p* = 0.01) was significantly more common and serum albumin at the time of device placement (*p* = 0.04) was significantly lower in two-session group, indicating that the two-session group may include more infirm patients than the single-session group. To allow for these possible confounding variables, multivariate analysis was conducted to control for any impact on early device infections.

G-tube infections are not uncommon. Minor peristomal G-tube infections have been reported to occur as many as 30% of cases. The early G-tube infection rates of the single-session group (7.1%) and two-session group (4.0%) are similar to the rate reported by other studies.^18,19^ Major G-tube infections that require aggressive medical and/or surgical treatment have been observed in less than 1.6% of patients.^18,20^ Two patients (1.0%) in the single-session group developed a major G-tube infection requiring intravenous antibiotics, which is within the reported range of patients requiring aggressive management. In these cases, the ports remained in place without signs of infection. Bacteremia associated with G-tube placement is quite rare; few case series using either endoscopic or fluoroscopic guidance have been reported.^21,22^ The infrequency of bacteremia after G-tube placement is one plausible reason why there was no significant difference in early port infections between the two groups.

All patients in this study received an intravenous prophylactic antibiotic. Prophylactic antibiotics for port placement were not advocated according to the SIR clinical practice guidelines^23^ or Centers for Disease Control and Prevention recommendations.^7^ SIR guidelines recommend a case-by-case approach for use of antibiotic prophylaxis for ports in immunocompromised patients. Some evidence supports the administration of prophylactic antibiotics for percutaneous radiologic G-tube placement to reduce the risk of peristomal infections in patients with HNC,^24^ but there is no consensus whether prophylactic antibiotics are necessary for all G-tube placements when the push technique is used.^23,25^ The impact of the prophylactic antibiotics on device infection, especially in the setting of single-session placement is unclear in this study. There are several limitations in this study, mostly due to its retrospective nature and relatively small sample size. Patients were not randomly assigned to the two groups since the study cohort was retrospectively collected. The two-session group could have included more infirm patients than the single-session group, given significant difference in inpatient status and serum albumin at the time of device placements. To allow for these differences, multivariate analysis was conducted. However, the statistical power to detect the differences between the two groups is significantly limited given the small sample size, particularly in the two-session group, as well as the low incidence of infectious events in this study. Additionally, the interval between port and G-tube placements as well as the order of the placements in the two-session group was not standardized, which might impact the incidence of the device infections. Finally, it would be important to assess patient satisfaction in both groups to help determine if single-session placement is a more favorable approach compared to two-session placement from the patients’ perspective.

In conclusion, this study did not identify a significant difference in the incidence of early port or G-tube infections between the single-session and two-session groups. The risk of early device infection in single-session placement appeared to be the same as the two-session placement, which may have been performed in more infirm patients. This study would serve as the first step for a prospective randomized controlled trial to validate single-session placement of the two devices in patients with HNC undergoing chemoradiation.
